# An isothermal CRISPR- based lateral flow assay for detection of *Neisseria meningitidis*

**DOI:** 10.1186/s12941-024-00688-1

**Published:** 2024-03-30

**Authors:** Dao Thi Huyen, Julien Reboud, Dao Thanh Quyen, Jonathan M. Cooper, Thirumalaisamy P. Velavan, Ngo Tat Trung, Le Huu Song

**Affiliations:** 1 Vietnamese – German Center for Medical Research (VG-CARE), 108 Military Central Hospital, Nr 1, Tran Hung Dao Street, Hai Ba Trung Dist., Hanoi, 10000 Vietnam; 2 James Watt School of Engineering, University of Glasgow, Glasgow, G12 8QQ UK; 3 Department of Molecular Biology, 108 Military Central Hospital, Hanoi, 10000 Vietnam; 4 Institute of Tropical Medicine, University of Tübingen, 72074 Tübingen, Germany; 5 Centre for Genetics Consultation and Cancer Screening, 108 Military Central Hospital, Hanoi, 10000 Vietnam

**Keywords:** LAMP, CRISPR-Cas, *N. Meningitidis*, Meningococcal serogroups, CSF

## Abstract

**Background:**

*Neisseria meningitidis* can cause life-threatening meningococcal meningitis and meningococcemia. Old standard microbiological results from CSF/blood cultures are time consuming. This study aimed to combine the sensitivity of loop-mediated isothermal nucleic acid amplification (LAMP) with the specificity of CRISPR/Cas12a cleavage to demonstrate a reliable diagnostic assay for rapid detection of *N. meningitidis*.

**Methods:**

A total of *n* = 139 samples were collected from patients with suspected meningococcal disease and were used for evaluation. The extracted DNA was subjected to qualitative real-time PCR, targeting capsular transporter gene (*ctrA)* of *N. meningitidis*. LAMP-specific primer pairs, also targeting the *ctrA,* were designed and the LAMP products were subjected to CRISPR/Cas12 cleavage reaction. the readout was on a lateral flow strip. Sensitivity, specificity, positive predictive value (PPV) and negative predictive value (NPV) of LAMP-CRISPR/Cas was compared with real-time PCR assays. The limit of detection (LOD) was established with serial dilutions of the target *N. meningitidis* DNA and calculated by Probit regression analysis.

**Results:**

Six LAMP assay-specific primers were developed targeting the *ctrA* gene of *N. meningitidis*, which is conserved in all meningococcal serogroups. The LAMP primers did not amplify DNA from other bacterial DNA tested, showing 100% specificity. The use of 0.4 M betaine increased the sensitivity and stability of the reaction. LAMP-CRISPR/Cas detected meningococcal serogroups (B, C, W). The assay showed no cross-reactivity and was specific for *N. meningitidis*. The LOD was 74 (95% CI: 47–311) *N. meningitidis* copies. The LAMP-CRISPR/Cas performed well compared to the gold standard. In the 139 samples from suspected patients, the sensitivity and specificity of the test were 91% and 99% respectively.

**Conclusion:**

This developed and optimized method can complement for the available gold standard for the timely diagnosis of meningococcal meningitis and meningococcemia.

**Supplementary Information:**

The online version contains supplementary material available at 10.1186/s12941-024-00688-1.

## Introduction

*Neisseria meningitidis* can cause meningococcal meningitis septicaemia, a life-threatening infection of the meninges characterized by rapid onset, high fever, headache, stiff neck, nausea, vomiting and severe aches or pain. *N. meningitidis* are transmitted by infectious droplets (respiratory and throat secretions). Meningococcal meningitis can lead to long-term consequences even with appropriate treatment. Up to 49.2% of survivors suffer from one or more long-term sequelae including behavioural and/or intellectual disorders (45%), hearing changes (6.7%), and gross neurologic deficits (14.3%) [1]. The most common serogroups causing disease in humans include A, B, C, W, X and Y, although this varies in different geographical regions. The highest incidence is observed in sub-Saharan Africa and cases there are often caused by serogroups A, W, and X [2]. Serogroup A was responsible for large epidemics in Africa, while serogroups B and C have been widespread in other geographical regions. In the Asia–Pacific region predominant serogroups of *N. meningitidis* are B, W and Y, although serogroups A and X are also observed [3].

In Vietnam, invasive meningococcal disease is life-threatening with a high mortality rate and severe sequelae, and is more common in young children < 5 years of age [4]. Based on the available data from surveillance system in Vietnam, the incidence rate in 2018 was 0.02 per 100,000 population [4]. Studies have estimated higher incidence rates in the military population, ranging from 0.22 to 2.67 between 2018 and 2021, the highest incidence was 3.33/100,000 soldiers in 2016 [5]. Although there is limited information on the specific distribution of serogroups, B and C have been the most common [4–6].

Most invasive meningococcal cases are diagnosed and treated in specialized hospitals and there is a narrow window of opportunity for clinical diagnosis and medical intervention between the onset of the first symptoms and severe disease or death. The diagnosis of meningococcal meningitis is usually confirmed using cerebrospinal fluid (CSF) testing for the presence of *N. meningitidis* [7]. These tests may include bacterial cultures, polymerase chain reaction (PCR) and serogrouping to determine the specific serogroup of the bacteria involved. Rapid diagnostic tests can help in early diagnosis and treatment initiation, but the range of applications and sensitivity of the tests are limited, especially when performed with limited resources [8]. Although more sensitive and specific nucleic acid tests (NATs) are available, they often cannot be employed rapidly or within communities, limiting their broader impact [9].

In this study, we built on our previous proof-of-concept work [10] and combined the sensitivity of loop-mediated nucleic-acid isothermal amplification (LAMP) with the specificity of the CRISPR/Cas12a cleavage to demonstrate a reliable diagnostic point-of-care assay for the detection of *N. meningitidis*. The test comprises of two steps: (1) amplification of the bacterial DNA with LAMP; and (2) detection of amplicons using CRISPR-mediated collateral reporters. LAMP can achieve a similar sensitivity to PCR within 60 min and without complicated thermocycling. However, some reports have pointed out that the LAMP carries the risk of false positive results, which may be due to non-specific amplification [11]. To limit the risks, we have combined LAMP with the CRISPR-Cas, which recognizes and cleaves DNA molecules in a sequence-specific manner, using mature crRNAs as guides for the DNase Cas12a to specifically cut the target nucleic acids [12]. Simultaneously, the trans-cleavage activity of Cas12a system was activated to cut single-strand DNA such as probes [13]. The probes are often labelled with a fluorophore and a quencher, so that a fluorescent signal is released upon cleavage. In another configuration, we labelled the probe with two components to generate a signal in the form of a sandwich on a lateral flow strip [14], which disappears when the probe is cleaved. We further validated the developed assay against available gold standard methods, demonstrating its reliability, reproducibility, and operational performances.

## Materials and methods

### Ethics statement

All samples used in this study were obtained from patients admitted to 108 Military Central Hospital (108-MCH), Hanoi, Vietnam. The study was approved by the institutional review board and an independent ethics committee of 108 Military Central Hospital Hanoi, Vietnam (108MCH-# 2723/HĐĐĐ- 25,062,020). Written informed consent was obtained from all study patients and/or their guardians. All samples used in this study were pseudo-anonymized prior to laboratory testing to de-identify the patients.

### Bacterial strains

Positive control DNA was extracted from *N. meningitidis* serogroup B *(MC58, #ATCC BAA-335). N. meningitidis serogroup C (M1628, #ATCC 13,102)* and *W135 (M-1574*, #ATCC 43,744) were also used to confirm the diagnosis. Furthermore, non-meningococcal *Neisseria* species, including *N. gonorrhoeae* (*F-18, #ATTC 49,226*), and *Escherichia coli* DH5α *(#18,258,012, Thermo Fisher Scientific Inc)* were also tested for cross-reactivity.

### Clinical CSF samples

All samples were collected from patients with suspected meningococcal disease (*n* = 139). Clinicians classified patients as having probable meningococcal disease based on clinical signs and symptoms, CSF parameters (such as white blood cell count, glucose and protein levels) and bacterial tests (such as CSF culture and/or latex agglutination test), and close contact with a meningococcal patient.

### Detection of ***N. meningitidis*** with Realtime-PCR

DNA was extracted from 200 μL saliva/oropharyngeal swabs/ CSF using the automated SACACE SYSTEM (*Nucleic acid extraction laboratory workstation SaMag-12™, Sacace Biotechnologies S.r.l*., *Italy*) with an elution volume of 100 μL. Real-time qualitative PCR targeting the capsule transporter gene *ctrA* of *N. meningitidis* was performed using the set of primers described in Table 1. Reactions were performed in volumes of 20 μL containing 1X QuantiTect Probe PCR (Qiagen, Hilden, Germany), 0.25 μM of each primer, 0.25 μM of probe and 5 μL of template DNA. Realtime PCR was performed in duplicates in *AriaMx* Real-Time PCR system (Agilent Technologies, Inc, Santa Clara, California, United States) as follows: initial denaturation at 95 °C for 15 min, followed by 45 cycles of denaturation at 95 °C for 15s, annealing at 60 °C for 60s when the fluorescence signal was measured. Samples were classified as negative if Ct values ≥ 40 or no amplification signal was detected.

**Table 1:** Primers and Probes employed for LAMP-CRISPR and Real-time PCR assay

	N. meningitidis gene target	Sequences of Primer and Probes (5’-3’)	Tm in °C
LAMP-CRISPR/Cas (Primers, gRNA and probes)	*ctrA-*F3	TCCGCGACAAAATATTTTGCTGC	70
	*ctrA-*B3	CCATTTATCCTGACGTTCTGCCG	70
	*ctrA-*LF	CACCGCACCCATAGACGTAA	67
	*ctrA-*LB	CGTGGTGTGTTTGTGTTCCG	69
	*ctrA-*FIP	ACCGATTTCTTGTGTTCTCCCCATGATTACCAATCCCT	63 − 60
	*ctrA-*BIP	GTATGGGCGGTTTGCAAGATCGCATTCCACCAATGGCGT	58–63
	*ctrA-sg*RNA	UAAUUUCUACUAAGUGUAGAUAGCCAGAGGCUUAUCGCUUUC	62
	ssDNA FAM-biotin	/5(6)-FAM/TTATTATT/3BIO/	-
	ssDNA FQ reporter	/5(6)-FAM/TTATTATT/3BHQ-1/	-
Real-time PCR	*ctrA*_F	GCT GCG GTA GGT GGT TCA A	58
	*ctrA*_R	TTG TCG CGG ATT TGC AAC TA	45
	*ctrA*_probe	/FAM/CAT TGC CAC GTG TCA GCT GCA CAT/TAMRA/	54

*ctrA*: capsule transporter gene of *N. meningitidis*

### Loop-mediated isothermal amplification (LAMP) assays for ***N. Meningitidis***

LAMP-specific primer pairs for *Neisseria meningitidis* were designed. Details of the primer are described in Table 1. Reactions were performed in reaction volumes of 20 μL containing 1x isothermal buffer II (New England Biolabs, Ipswich, MA), 1.6 μM each FIP and BIP, 0.2 μM each F3 and B3, 0.4 μM each LF or LB, 0.25 μM dNTPs, 2.56 U of *Bst*3.0 DNA polymerase (New England Biolabs, Ipswich, MA), 0.4 M betaine (Sigma, St. Louis, MO), a truncated gene 2.5 protein (GP2.5-delta 21 C) and 5 μL of template DNA. LAMP reactions were performed in heat blocks (Block Thermostat BT200, Kleinfeld Labortechnik, Germany) at 63 °C for 20 min followed by an inactivation step for 5 min at 80 °C.

### CRISPR/Cas12 cleavage reaction

Detection assays were performed using 1x r2.1 buffer, 25 nM LbCas12a (New England Biolabs, Ipswich, MA), 25 nM gRNA, 750nM custom synthesized homopolymer ssDNA FQ reporter or 125nM ssDNA FAM-biotin reporter (see Table 1). All these components were mixed within 20 μL and added directly into LAMP reaction tube (total volume 40 μL). It was incubated at 42 °C for 40 min. Finally, the entire volume was applied to lateral flow (PCRD, Abingdonhealth – FG-FD51673) for “naked-eye” detection. If the strip shows two bands (T2 and C lines), the result is negative. If it shows one band (C band line), the result is positive.

### ***Neisseria meningitidis*** dilution series to determine the limit of detection (LOD)

The limit of detection (LOD) has been calculated as the lowest amount of analyte, which can be detected with more than a stated percentage of confidence, but not necessarily quantified as an exact value [15, 16]. In molecular assays, the LoD value is generally considered to be the lowest concentration of the target that can be detected in ≥ 95% of replicate measurements [17]. In this study, multiple aliquots of a specific matrix were spiked with serial dilutions of the target DNA and subjected to the entire process of LAMP-CRISPR/Cas testing. The LOD is then defined as the spike amount of target DNA in dilution that could be detected in 95% of replicates [18]. DNA extraction from colonies of *N. meningitidis* type B standard strains (MC58). DNA concentration was converted to copy number using a genome length for *N. meningitidis* type B standard strains (MC58) of 2,272,351 bp. LOD was calculated by probit regression analysis with 20 repeats for each level.

### Statistical analysis

Sensitivity, specificity, positive predictive value (PPV), and negative predictive value (NPV) of real-time PCR tests were compared with those of LAMP-CRISPR/Cas (real-time PCR was considered the gold standard). Probit regression analysis was performed using SPSS software ver. 20 (IBM Armonk, New York United States).

## Results

### CRISPR/Cas- LAMP combination assay

**Fig. 1:**
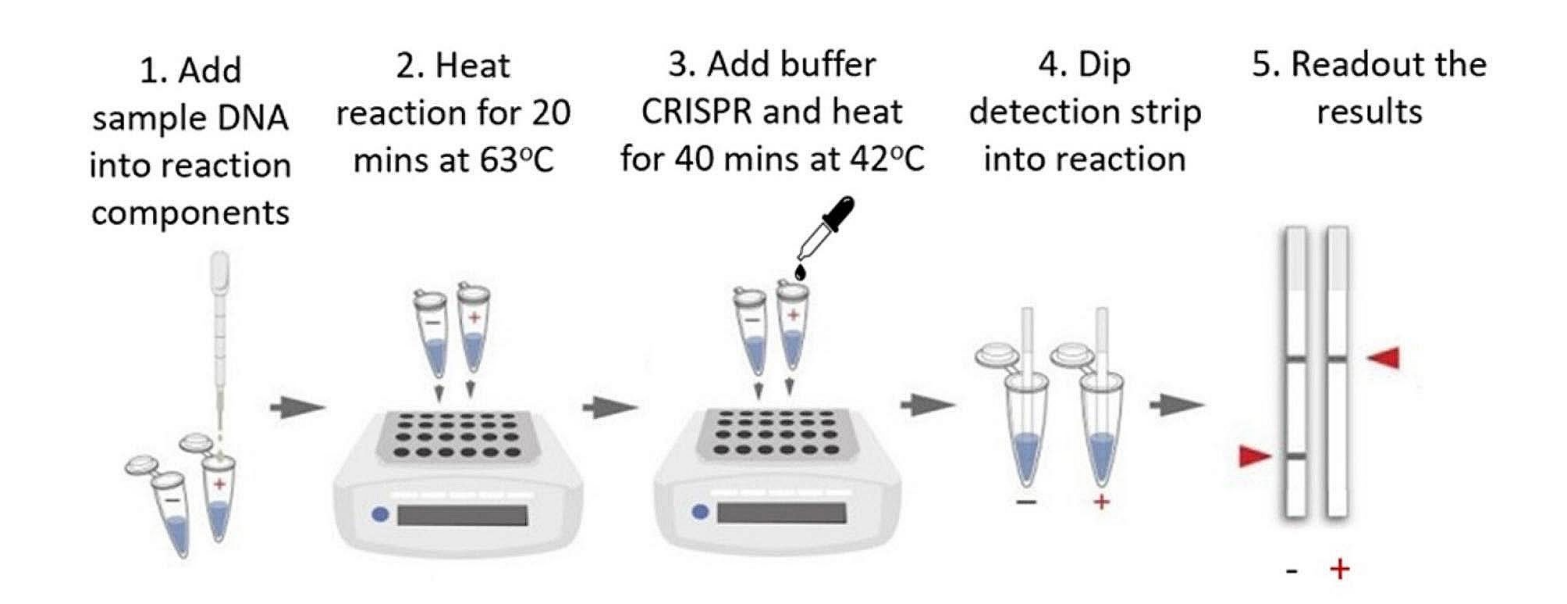
Schematic overview of the lateral flow readout assay for the molecular detection of N. meningitidis by CRISPR-Cas cleavage of LAMP amplicons

**Fig. 2:**
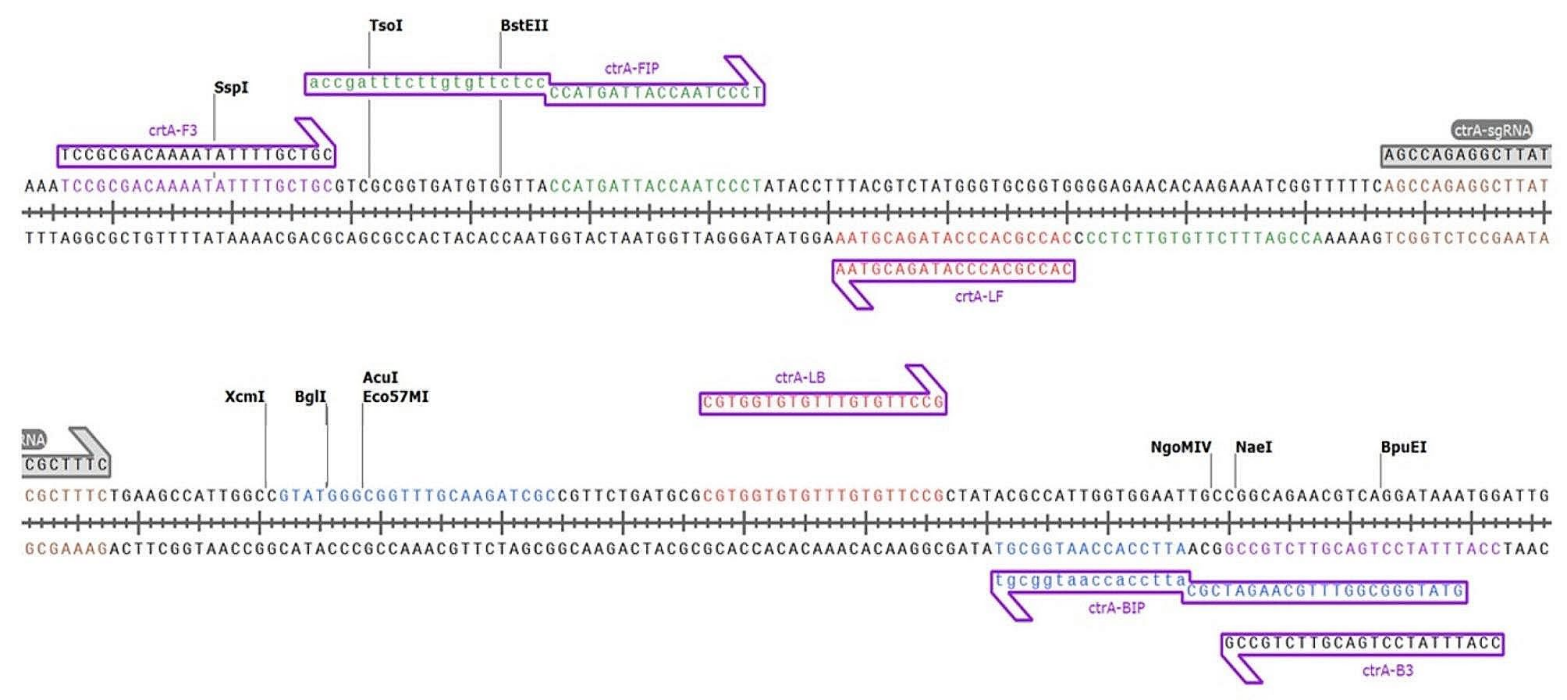
LAMP assay using six primers that are highly conserved in the meningococcal serogroups and specifically detect the *N. meningitidis* ctrA gene, which encodes an outer membrane protein that regulates capsular transportation

**Fig. 3:**
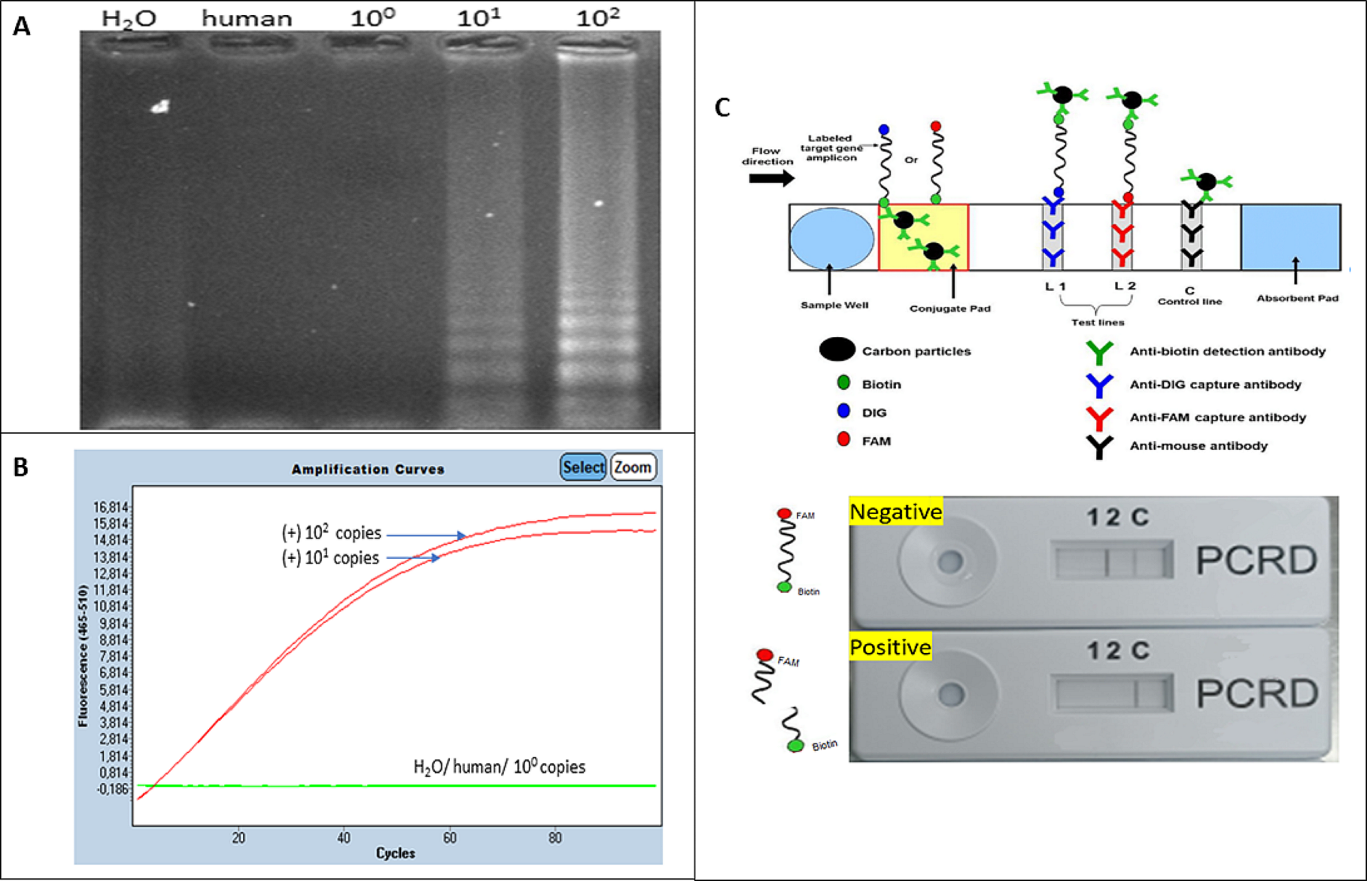
(**A**) *N. meningitidis*-specific LAMP assay amplicons visualized in an agarose gel (HO: negative control; human DNA: negative control; lanes 3–5 show the tested N. meningitidis concentrations 10^0^: zero copies/ μL; 10^1^: ten copies/ μL; 10^2^: 100 copies/ μL). (**B**) Fluorescent signals detected by real-time PCR after CRISPR/Cas cleavage (red colour indicates 10 and 100 copies); green colour indicates human DNA and water as negative controls and 10^0^: zero copies/μL). (**C**) Readout from the lateral flow assay. The test cassette shows two lines for negative samples (un cleaved) and one line for positive samples (cleaved)

The overall process of the lateral flow readout assay for the molecular detection of *N. meningitidis* by CRISPR-Cas cleavage of LAMP amplicons has been illustrated (Fig. 1). The LAMP assay using six primers that are highly conserved in the meningococcal serogroups [19] and specifically detect the *N. meningitidis ctrA* gene, which encodes an outer membrane protein that regulates capsular transportation has been illustrated (Fig. 2). The *N. meningitidis*-specific LAMP assay amplicons as visualized in an agarose gel and the corresponding fluorescent signals detected after CRISPR/Cas cleavage by real-time PCR are shown (Fig. 3A and B). The salt concentration had no effect on the sensitivity and specificity of the LAMP reactions (see Supplementary Figure S1A). We also optimized other components of the buffer (including the concentrations of betaine, sorbitol, DMSO, glucose, single-stranded DNA binding protein). The result was that 0.4 M betaine with a single-stranded DNA binding protein (GP2.5-delta 21 C) increased the sensitivity and stability of the reaction (see Supplementary Figure S1B, S1C). The *N. meningitidis* with 10 copies per reaction were detected within 20 min (see Supplementary Figure S1D), which was consistent with the study as described by Lee et al. [20]. The fluorescent signals after CRISPR reaction clearly show the positive samples without false positives, even when excess DNA from human cells is used. To simplify the read-out and allow the assay to be performed outside of well-equipped laboratories, we also used a FAM biotin probe that allows the assay to be performed on a lateral flow strip. The readout results of the lateral flow assay in a test cassette with negative (non-cleaved) and positive (cleaved) samples are described (Fig. 3C). Our results showed that LAMP-CRISPR/Cas combination can detect *N. meningitidis* serotype C *(M1628, #ATCC 13,102)* and W135 *(M-1574, #ATCC 43,744*) as well (see Supplementary Figure S2A). The results of the alignment show a 100% match of the target DNA sequence when compared to other strains using the nucleotide BLAST tool (see Supplementary Figure S2B).

### Optimization and limit of detection (LOD) of CRISPR/Cas- LAMP assay

We optimized the assay with a lateral flow readout using *N. meningitidis* genome standards spiked with human genome to determine the LOD, incubation temperature, readout time, and robustness. The ideal incubation parameters were 63 °C for 20 min for LAMP and 42^o^C and at least 40 min for CRISPR-Cas cleavage and lateral flow processing. The LOD value of the method was determined by a probit regression analysis with 20 replicates for each stage. The LOD was 74 *N. meningitidis* copies with 95% CI: 47–311 (see raw data in supplementary Table S1) and analysed data as shown in Table 2.

**Table 2:** Limit of detection using Probit regression analysis for the combined CRISPR/Cas-LAMP assay. (Values indicated in bold are the LOD was 74 *N. meningitidis* copies

Probability	95% Confidence limit		
	Estimate	Lower Bound	Upper Bound
0.010	-102.578	-211.139	-61.796
0.020	-90.449	-188.779	-53.383
0.030	-82.754	-174.605	-48.033
0.040	-76.965	-163.950	-44.000
0.050	-72.257	-155.289	-40.714
0.060	-68.249	-147.922	-37.912
0.070	-64.735	-141.466	-35.451
0.080	-61.588	-135.690	-33.244
0.090	-58.727	-130.440	-31.233
0.100	-56.093	-125.610	-29.380
0.150	-45.187	-105.652	-21.667
0.200	-36.519	-89.848	-15.478
0.250	-29.083	-76.350	-10.109
0.300	-22.405	-64.296	-5.220
0.350	-16.217	-53.206	− 0.610
0.400	-10.345	-42.784	3.867
0.450	-4.664	-32.835	8.333
0.500	0.927	-23.234	12.917
0.550	6.518	-13.910	17.779
0.600	12.199	-4.858	23.142
0.650	18.070	3.855	29.327
0.700	24.258	12.112	36.771
0.750	30.936	19.863	45.963
0.800	38.372	27.298	57.395
0.850	47.040	34.918	71.767
0.900	57.946	43.633	90.723
0.910	60.580	45.651	95.388
0.920	63.442	47.817	100.483
0.930	66.588	50.173	106.111
0.940	70.102	52.776	112.424
**0.950**	**74.110**	**55.715**	**119.654**
0.960	78.819	59.136	128.181
0.970	84.608	63.304	138.701
0.980	92.303	68.795	152.734
0.990	104.431	77.369	174.933

### CRISPR/Cas- LAMP assay specificity

**Fig. 4:**
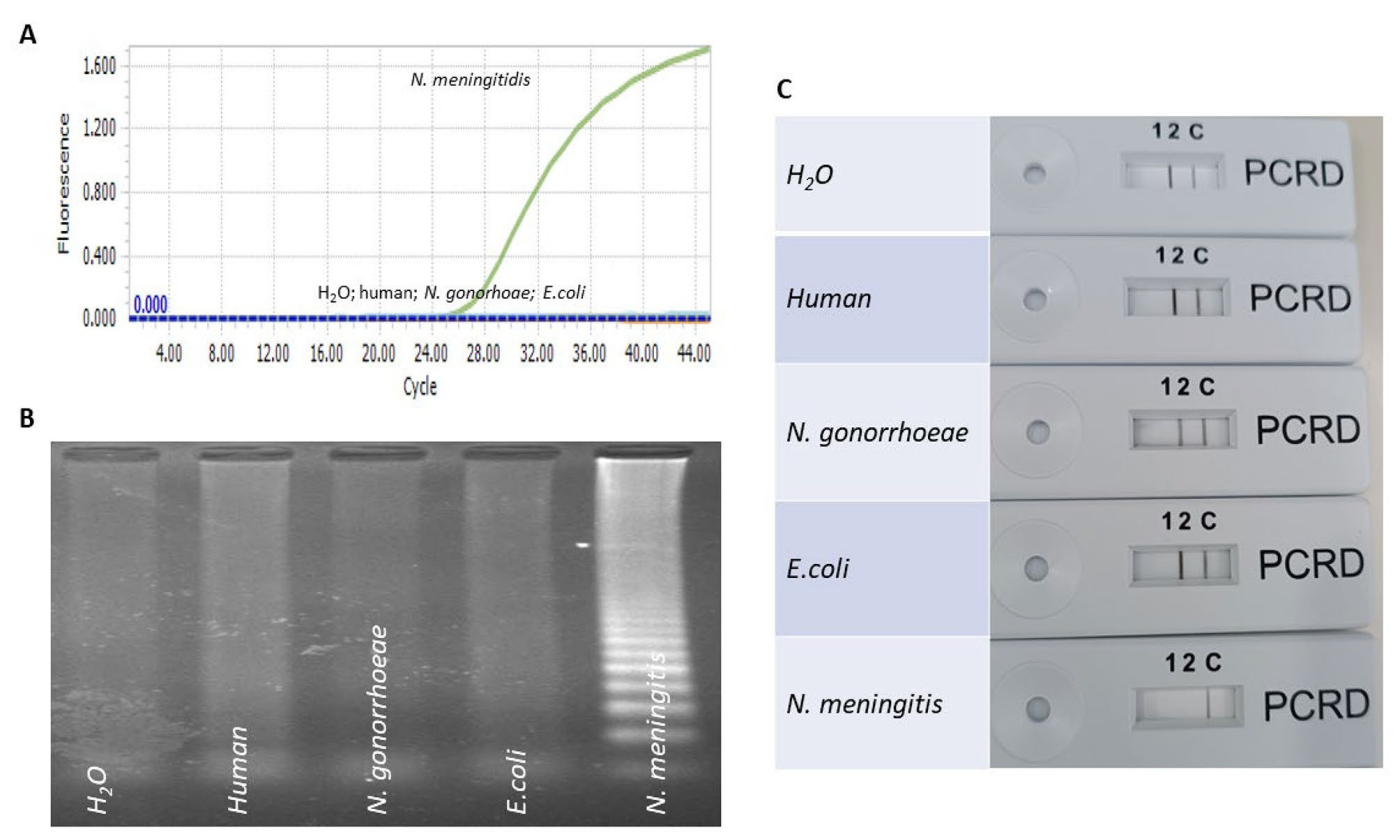
(**A**). Real-time PCR detection of *N. meningitidis*; (**B**). LAMP amplicons visualized on agarose gel (lane1: water (H20), lane 2: human DNA (20ng), lane 3: *N. gonorrhoeae* DNA (10^3^ copies), lane4: *E. coli* DNA (10^3^ copies ), lane 5: *N. meningitidis* DNA (10^3^ copies). (**C**). Readout from the lateral flow assay. The test cassette shows two lines for all negative samples (un cleaved) and one line for *N. meningitidis* positive sample (cleaved)

The assay was applied to DNA extracted from (1) *Neisseria meningitidis* type B (*MC58*), (2) non-meningococcal *Neisseria* species including *N. gonorrhoeae* (NIID9), and (3) *Escherichia coli*. The LAMP primers did not amplify DNA from other bacterial DNA tested (100% specificity). The assay exhibited no cross-reactivity and was specific for *N. meningitidis* (Fig. 4).

### CRISPR/Cas- LAMP assay evaluation clinical samples with real-time PCR

Using the optimized LAMP-CRISPR/Cas assay, 139 clinical CSF samples were tested for *N. meningitidis*. All samples were independently validated using the gold standard real-time PCR method. Both methods detect the same *ctrA* gene as a target. The results of the two methods were compared. The diagnostic accuracy (sensitivity and specificity) of the *N. meningitidis* test based on the LAMP-CRISPR/Cas12 assay was calculated with reference to the real-time PCR results (Table 3). The combined LAMP-CRISPR/Cas assay had a sensitivity of approximately 91% and a specificity of 99% (Table 3). Real-time PCR-positive samples with Ct values ≤ 27 were detected by LAMP-CRISPR/Cas within 60 min (see Supplementary Figure S3A). False negative LAMP-CRISPR/Cas samples (*n* = 5) had qPCR average Ct values = 35 (see Supplementary Figure S3B), which corresponds to a load of less than 10 copies and is consistent with the LOD. The kappa agreement index (ĸ) between the two methods reached 0.909 (a statistical analysis in crosstabs statistics) and thus a high degree of agreement.

**Table 3:** Positive and negative predictive value, sensitivity and specificity of LAMP-CRISPR/Cas for the detection of *N. meningitidis* in clinical samples

LAMP-CRISPR/Cas	Real-time PCR			PPV	NPV	Sensitivity	Specificity
	Positive	Negative	Total	Number/ Total number (%)			
Positive	50	1	51	50/51 (98.04)		50/55 (90.91)	
Negative	5	83	88		83/88 (94.31)		83/84 (98.81)
Total	55	84	139				

PPV: positive predictive value; negative predictive value (NPV)

## Discussion

Invasive meningococcal disease can be severe and include infections of the brain and its membranes (meningococcal meningitis) and the bloodstream (meningococcemia). While diagnosis often involves analysis of blood or CSF samples to detect the bacteria by microbiological isolation of *N. meningitidis*, shorter turnaround time and improved diagnostics are critical for timely clinical management, particularly in resource-limited settings where rapid and decentralized testing is paramount. As the landscape of diagnostics is dynamic and new technologies are constantly emerging, faster, adequate molecular diagnostics can support and complement the early detection and timely management of meningococcal meningitis and meningococcemia while waiting for the gold standard microbiological results from CSF/blood culture.

While several biomarkers can support the diagnosis, the nucleic acid test (NAT) is the gold standard for various acute infectious diseases. In our method, we have combined the sensitivity of loop-mediated isothermal nucleic acid amplification (LAMP) with the specificity of CRISPR/Cas12a cleavage to demonstrate a reliable diagnostic point-of-care assay for the detection of *N. meningitidis*. The clinical use of CRISPR system for pathogen diagnosis is often hampered by the low copies of the pathogen. However, this study supported the introduction of a nucleic acid pre-amplification step by LAMP to achieve a clinically relevant limit of detection (LoD). In this study, we used the LAMP assay amplification method PCR, which does not require expensive thermal cycler or trained personnel. We also found that the addition of 0.4 M betaine with the GP2.5-delta 21 C protein increased the sensitivity and stability of the LAMP reaction and enabled a shorter reaction time (20 min). In addition, the specific cleavage by CRISPR/Cas eliminated the non-specific amplification visible to the naked eye. With an LOD of 74 copies with a sensitivity of 91% and a specificity of 99%, the LAMP combination of CRISPR/Cas is performed well with gold standard methodologies.

Overall, our aim is to create diagnostic assay that fulfil the ASSURED criteria (affordable, sensitive, specific, user-friendly, rapid, device-free, delivered) set by the World Health Organisation (WHO) [21]. This simplified format of LAMP-CRISPR/Cas is a reliable, sensitive, specific, user-friendly, rapid and instrument-free method that can be implemented in centralized clinical laboratories. Although the designed primers are conserved in all serogroups (A, B, C, W and Y), we were able to show that the primers are specific for serogroups B, C and W and could be detected in reference strains carrying the respective serotypes.

Although the LAMP assay has several advantages, such as simplicity and fast turnover time, there are certain limitations that make it less suitable for field diagnostics. For example, the LAMP assay is susceptible to non-specific amplification from environmental contaminants or impurities that may be present in the samples. Furthermore, . CRISPR-Cas assays often require complex sample preparation procedures, including DNA extraction, which can be cumbersome in field environments without access to well-equipped laboratories. Obtaining regulatory approval for field use could be a barrier to the widespread application of this technology in certain areas.

## Conclusion

Taken together, we have developed a LAMP-CRISPR/Cas assay for diagnosis of *N. meningitidis* and validated it against the available gold standard methods, demonstrating its reliability, reproducibility and operational performance. The assay can be implemented in centralized laboratories complementing gold standard methodologies.

### Electronic supplementary material

Below is the link to the electronic supplementary material.

Supplementary Material 1

## Data Availability

No datasets were generated or analysed during the current study.
