# Supplementary material for: An isothermal CRISPR- based lateral flow assay for detection of Neisseria meningitidis
Source: Ann Clin Microbiol Antimicrob. 2024 Mar 30;23:28. doi: 10.1186/s12941-024-00688-1 (PMC10981803; doi:10.1186/s12941-024-00688-1)

# An isothermal CRISPR- based lateral flow assay for detection of *Neisseria meningitidis*

**Supplementary file**

Table S1. Results were repeatable at different concentrations of *Neisseria meningitidis* (6 copies; 8 copies; 10 copies; 25 copies; 50 copies; 75 copies and 100 copies). Count observed responses (positive) at each time of 20 repeats

| **Cell Counts and Residuals** | | | | | | | |
| --- | --- | --- | --- | --- | --- | --- | --- |
|  | Number | Copies number | Total of Subjects | Observed Responses | Expected Responses | Residual | Probability |
| PROBIT | 1 | 6.000 | 20 | 6 | 10.908 | -4.908 | 0.545 |
|  | 2 | 8.000 | 20 | 15 | 11.263 | 3.737 | 0.563 |
|  | 3 | 10.000 | 20 | 13 | 11.616 | 1.384 | 0.581 |
|  | 4 | 25.000 | 20 | 15 | 14.115 | 0.885 | 0.706 |
|  | 5 | 50.000 | 20 | 16 | 17.300 | -1.300 | 0.865 |
|  | 6 | 75.000 | 20 | 19 | 19.041 | -.041 | 0.952 |
|  | 7 | 100.000 | 20 | 20 | 19.740 | 0.260 | 0.987 |

**Figure S1.** (A) Survey MgSO4 at different concentrations between 2mM to 8mM (B) Survey at different concentrations 0.2M – 0.4M – 0.6M – 0.8M betain; (C) combine with GP2.5-delta 21C or increase Mg2+ to 6mM or add in 0.1 mg/ml BSA; (D) LAMP detects dilution series of N. meningitidis; NC mean only H2O as negative control, Human DNA as negative control also, PC-1 with 101 copies of *N. meningitidis*, PC-2 have 102 copies of *N. meningitidis.*

| 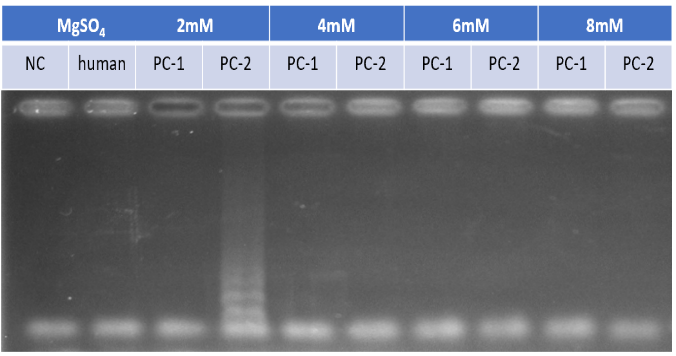 |
| --- |
| 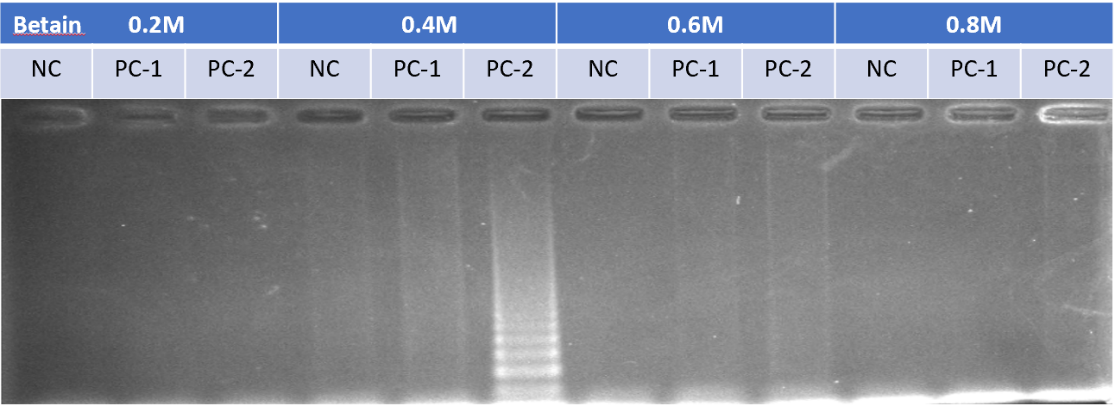 |
| 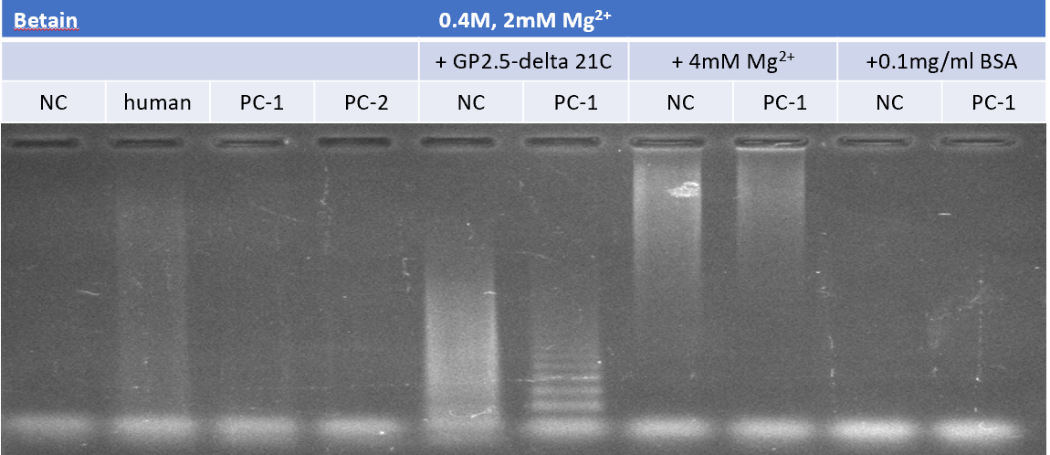 |
| 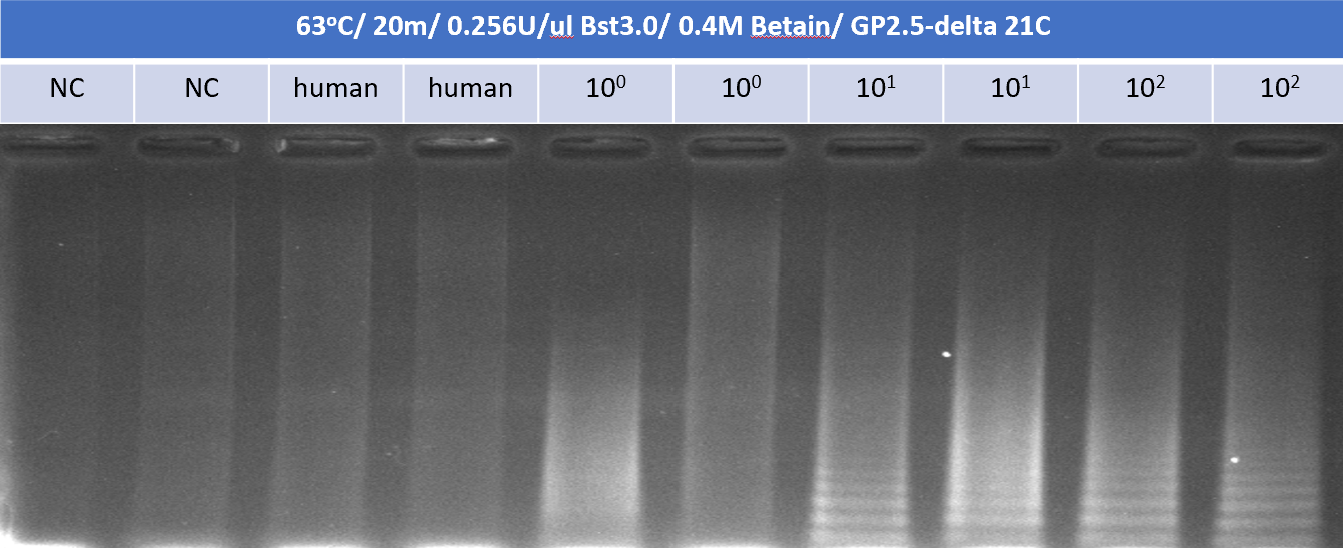  **Figure S2**. Amplified products of Neisseria meningitidis serogroup B (MC58, #ATCC BAA-335) - 10^3^ copies, C (M1628, #ATCC 13102) - 10^3^ copies and W135 (M-1574, #ATCC 43744) - 10^3^ copies confirmed by Loop-mediated isothermal amplification (LAMP) (A); by Sanger sequencing (B).   \| A.  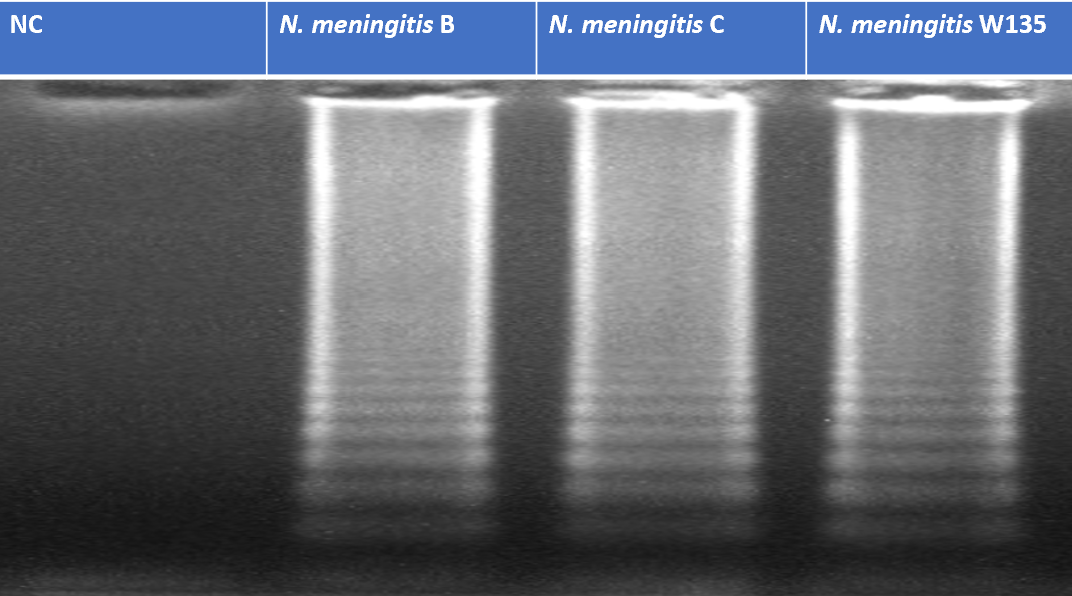 \| \| --- \| \| B.  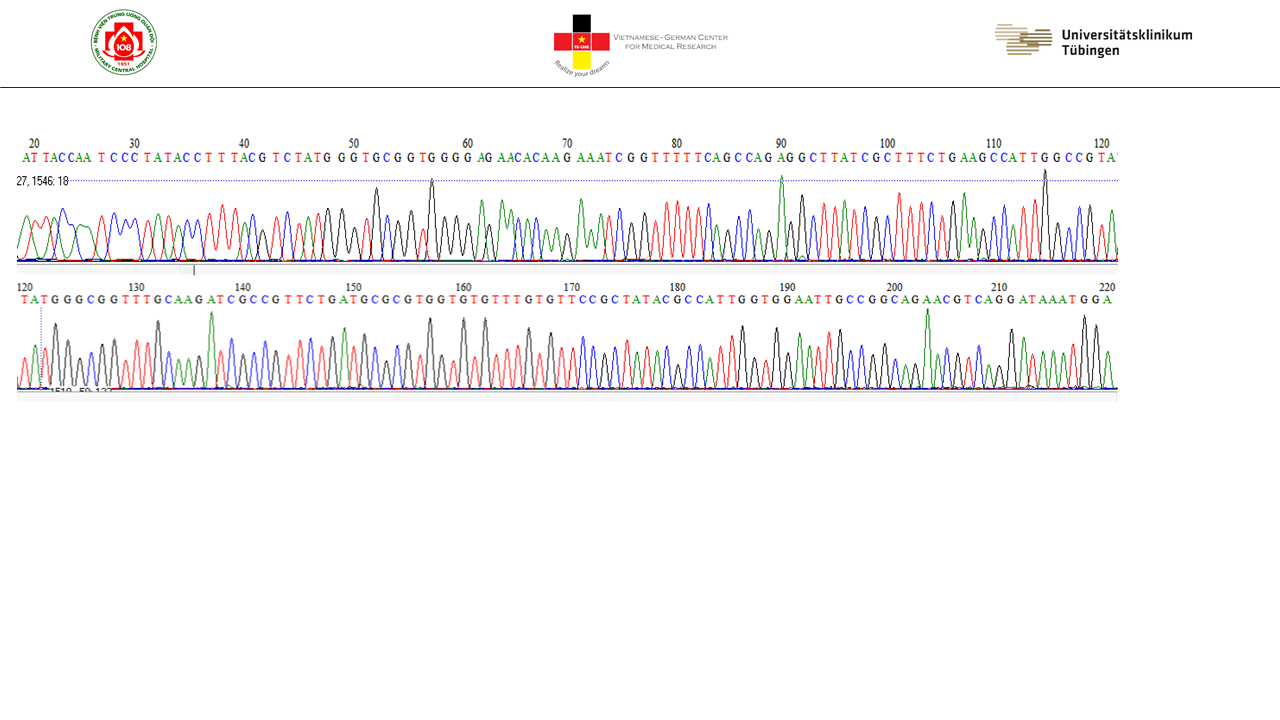 \| |

**Figure S3.** Ct value of positive samples detected by real-time PCR. (A) Ct value of positive sample confirmed by LAMP-CRISPR/Cas; (B). Ct value of the 5 positive samples that were missed by the LAMP-CRISPR/Cas assay.


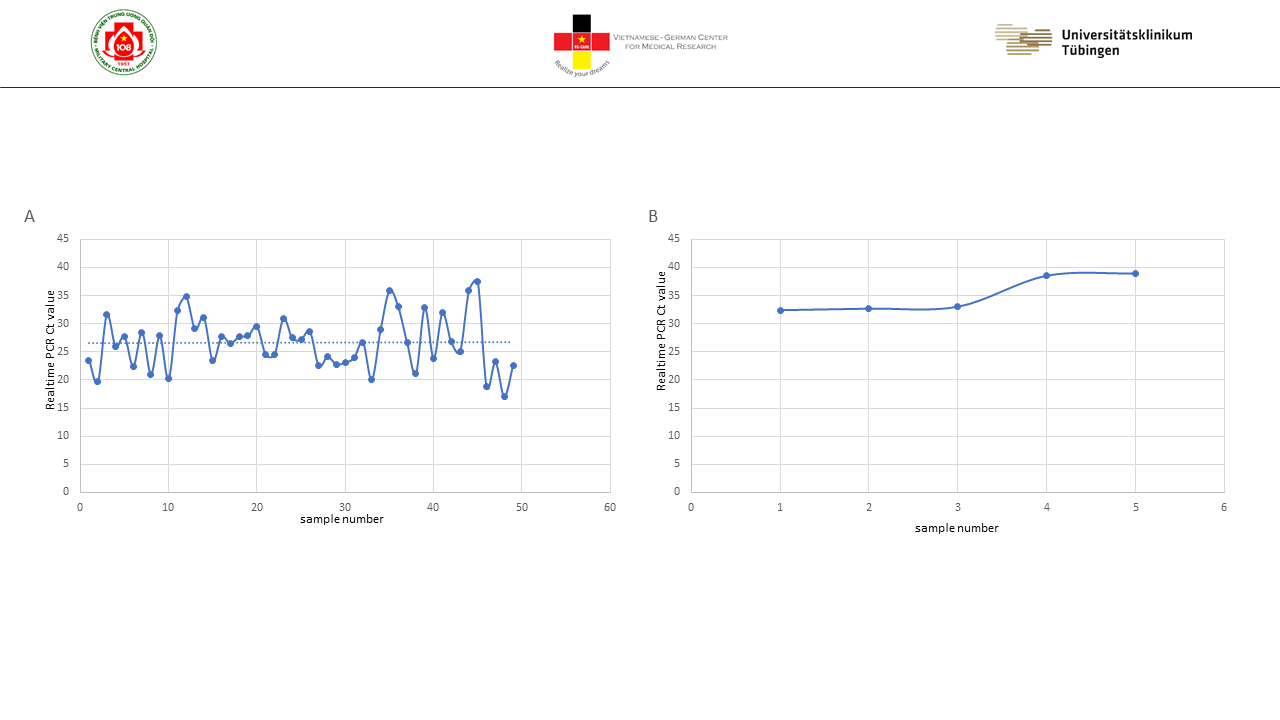

Supplement: Supplementary file 1 — Supplementary Material 1 [file 12941_2024_688_MOESM1_ESM.docx]
